# Myeloid cells in tumor inflammation

**DOI:** 10.1186/2045-824X-4-14

**Published:** 2012-09-03

**Authors:** Michael C Schmid, Judith A Varner

**Affiliations:** 1 Moores UCSD Cancer Center, University of California, San Diego, 3855 Health Sciences Drive, La Jolla, CA, 92093-0912, USA

**Keywords:** Macrophage, Myeloid derived suppressor cells, Tumor angiogenesis, Tumor microenvironment, Tumor inflammation, Cancer

## Abstract

Bone marrow derived myeloid cells progressively accumulate in tumors, where they establish an inflammatory microenvironment that is favorable for tumor growth and spread. These cells are comprised primarily of monocytic and granulocytic myeloid derived suppressor cells (MDSCs) or tumor-associated macrophages (TAMs), which are generally associated with a poor clinical outcome. MDSCs and TAMs promote tumor progression by stimulating immunosuppression, neovascularization, metastasis and resistance to anti-cancer therapy. Strategies to target the tumor-promoting functions of myeloid cells could provide substantial therapeutic benefit to cancer patients.

## Inflammation and cancer

Chronic inflammation is a causative or exacerbating factor in a host of complex human diseases, including solid tumors and leukemias/lymphomas, chronic bacterial and parasitic infections, rheumatoid arthritis, Crohn’s disease, asthma and central nervous system (CNS) disorders such as Alzheimers’ disease, Parkinson’s disease and multiple sclerosis. In each of these diseases, affected tissues are heavily invested with inflammatory myeloid cells, which include resident or bone marrow derived macrophages [1-4]. In addition, all tumors are heavily invested with myeloid cells, including tumor-associated macrophages (TAMs) [5,6]. Myeloid cells stimulate cancer initiation, malignant progression, metastasis and resistance to therapy [7]. Thus, targeting molecular pathways regulating the tumor promoting functions of myeloid cells holds promise for solid tumor therapy.

## Macrophages in normal and tumor biology

Macrophages are myeloid lineage cells that arise from bone marrow derived monocytic progenitor cells that differentiate into tissue macrophages, antigen-presenting dendritic cells and bone resorbing osteoclasts [8,9]. Macrophages can be activated in response to environmental signals, including microbial products and cytokines. Activated macrophages can be loosely divided into M1 (classically activated) and M2 (alternatively activated) phenotype [1]. Classical activation occurs in response to bacterial moieties such as lipopolysacharide (LPS) and immune stimuli such as interferon γ (IFNγ). M1 macrophages mediate resistance against intracellular parasites and tumors and elicit tissue disruptive reactions by secreting tumoricidal agents such as tumor necrosis factor α (TNF-α), interleukin-12 (IL-12), and reactive nitrogen and oxygen intermediates (RNI, ROI). In addition, M1 macrophages promote T-helper-l (Thl) responses. In general, M2 macrophages exhibit an immunosuppressive phenotype and release factors that include IL-l0 and Arginase-1 [10,11].

M2 macrophages are the predominant type of macrophage found in tumors [6]. M1 macrophages are abundant at sites of chronic inflammation and in early tumors [12,13], but then switch to an M2-like phenotype during tumor progression [14-16]. Although IL-4, IFNγ, and several other tumor-derived cytokines and growth factors modulate macrophage phenotypes *in vitro* and *in vivo*[1,17], the molecular mechanisms that promote M1 or M2 TAM subsets within the tumor microenvironment are incompletely understood.

Although TAMs can convert into M1 or M2 phenotypes, and thereby execute almost diametrically opposed biological functions, unique cell surface markers that distinguish the two TAM phenotypes remain elusive. Flow cytometric analysis does indicate that M1-like TAMs express an F4/80 + CD11c^+^MRC^low^ phenotype, while M2-like TAMs express an F4/80 + CD11c^neg^MRC^high^ phenotype [18].

## Myeloid derived suppressor cells

Myeloid derived suppressor cells (MDSC) are CD11b^+^Gr1^+^ immunosuppressive, incompletely differentiated myeloid progenitor cells originally identified in tumor bearing mice [19]. MDSC accumulate in the blood, spleen, lymph nodes, bone marrow and tumors of tumor-bearing animals and patients [20-24]. MDSCs inhibit innate and adaptive immunity, promoting tumor immune escape. MDSC are a heterogeneous population of cells that lack the expression of cell surface markers that are specifically expressed on macrophages or DC [25]. In mice, MDSC are uniformly characterized by the expression of Gr1 and CD11b. Gr1 includes the macrophage and neutrophil markers Ly6C and Ly6G, respectively, whereas CD11b (also known as integrin αM) is characteristic for the myeloid- cell linage. In recent years, several other surface molecules have been used to identify additional subset of immunosuppressive MDSC, including CD80 [26], CD115 (also known as macrophage colony stimulating factor (M-CSF) receptor) and CD124 (IL-4 receptor alpha chain, IL-4Rα) [27]. In addition, nuclear morphology has also been used to characterize mouse MDSC. Mononuclear CDllb^+^Gr1^mid^Ly6G^+/−^Ly6C^high^CD49d^+^ cells are considered “monocytic” whereas polymorphonuclear CDllb^+^Gr1^high^Ly6G^+^Ly6C^neg^CD49d^neg^ MDSC are considered granulocytic [28-30]. Subpopulations of MDSC can give rise to CD11b^+^Gr1^low^F4/80^+^MHCII^+^ macrophages with potent immunosuppressive properties, underscoring the potential biological continuum of immature myeloid cells, monocytes, and macrophages [20,25,31].

In patients with glioblastoma, breast cancer, colon cancer, lung cancer or kidney cancer, MDSC have been defined as Lin^neg^CDllb^+^HLA-DR^neg^CD33^+^ cells that express the common myeloid marker CD33 but lack mature monocyte and lymphoid cell linage markers (Lin^neg^ = CD14^neg^, CD3^neg^, CD19^neg^) and lack the MHC class II molecule HLA-DR [32]. In patients with renal cancer, polymorphonuelcar MDSC have been shown to express CD11b^+^ CD14^neg^CD15^+^CD66b^+^ VEGFR1^+^[33] whereas in patients with melanoma, prostate cancer, hepatocellular carcinoma or head and neck cancer, immunosuppressive monocytic CD11b^+^ CD14^+^ HLA-DR^low/neg^ MDSC were found [21,34-36]. These cells are associated with increased tumor burden and poor prognosis in patients with breast and colorectal cancer [24,37].

## Mechanisms of myeloid cell recruitment

Immune cell trafficking *in vivo* is regulated by chemokines and cytokines, and by members of the integrin, immunoglobulin and selectin adhesion molecule families [38,39]. A diverse array of chemotactic factors that are expressed either by tumor cells or tumor infiltrating immune cells stimulate myeloid cell recruitment into tumors. These factors include CCL2 (MIP-1), CCL5 (RANTES), CCL12, IL-8, IL-lβ, CXCL12 (SDF-1α), and CXCL5 (ENA-78) [40-44].

While malignant tumor cells express myeloid cell chemoattractants, tumor infiltrating immune cells also express a variety of chemotactic factors, which can further foster myeloid cell recruitment and accumulation in the tumor microenvironment. For example, myeloid cell derived IL-1β stimulates myeloid cell recruitment *in vivo* and pharmacological inhibition of IL-1β reduced the infiltration of myeloid cells into the tumor microenvironment and inhibited tumor progression in a lung cancer tumor model [42]. Tumor derived factors, such as G-CSF, can also stimulate long-range effects in the bone marrow, leading to myeloid cell expression of Bv8, a factor that stimulates myelopoiesis and mobilization [45,46].

Recent efforts have also been made to identify tumor-derived factors that specifically recruit myeloid cells in response to chemotherapeutic treatments. CCL2 and CCL12 were highly upregulated in doxorubicin treated MMTV-PyMT animals; genetic depletion of CCR2 or pharmacological blockade of GPCR-mediated signaling with pertussis toxin, reduced myeloid cell recruitment in response to chemotherapy and increased the sensitivity of tumors [41]. Paclitaxel treatment of MMTV-PyMT animals induced colony stimulating factor 1 (CSF-l) and IL-34 expression, which together stimulated CSF1 receptor (CSF1R)-dependent macrophage infiltration [47]. Blockade of CSF1R signaling in combination with paclitaxel improved survival of mammary tumor-bearing mice. Myeloid cells thus play a central role in resistance to chemotherapy.

## Roles of integrins in myeloid cell recruitment

The integrin adhesion molecule family is an extensive group of structurally related receptors for extracellular matrix (ECM) proteins and immunoglobulin superfamily molecules. Integrins are divalent cation-dependent heterodimeric membrane glycoproteins comprised of non-covalently associated α and β subunits that promote cell attachment and migration on the surrounding extracellular matrix. Eighteen α and eight β subunits can associate to form twenty-four unique integrin heterodimers [48,49]. Integrins on bone marrow-derived immune cells promote tumor inflammation by facilitating myeloid cell trafficking to the tumor microenvironment [42,50,51]. Myeloid cells express a number of functional integrins, including α2β1, α4β1, α5β1, αvβ3, αvβ5, αMβ2 (CD11b) and αXβ2 (CD11c) [52-54]. Recent studies from our laboratory indicate that integrin α4β1, a receptor for vascular cell adhesion molecule 1 (VCAM-1) and CS-l fibronectin, selectively promotes the homing of myeloid cells to the tumor microenvironment [42,55]. Human and murine myeloid cells adhered to endothelial cells *in vitro* and to tumor endothelium *in vivo* via integrin α4β1. Genetic and pharmacological blockade of integrin α4βl significantly suppressed tumor inflammation, growth and metastasis. In addition, combination of anti-integrin α4 antibody and chemotherapeutic agents markedly reduced tumor burden compared to chemotherapeutic treatment alone [42]. Thus, these studies indicate that suppression of myeloid cell trafficking to the tumor microenvironment with integrin α4βl antagonists could be a useful adjuvant approach in cancer therapy.

## Signaling molecules in myeloid cell recruitment

**Figure 1:**
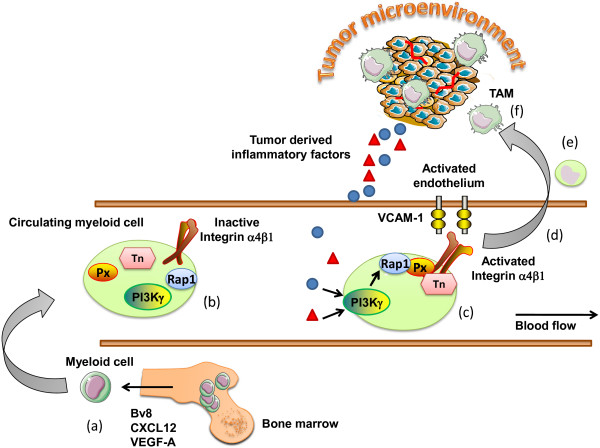
**Mechanisms regulating the recruitment of myeloid cells to the tumor microenvironment.** (**a**) Myeloid cells are activated and mobilized from the bone marrow in response to tumor derived factor such as Bv8, SDF-1α, GM-CSF. (**b**) Circulating myeloid cells express integrin adhesion molecules in an inactive confirmation, with low binding affinity. (**c**) In response to diverse chemotactic factors released from the tumor microenvironment, myeloid PI3Kγ activates the adhesion molecule integrin α4β1. (**d**) Myeloid cells are now able to bind to the activated tumor endothelium expressing the integrin α4β1 ligand VCAM-1. (**e**) Myeloid cells extravasate from the blood stream and migrate toward the tumor microenvironment, (**f**) where they differentiate in response to the cytokines/chemokine milieu to tumor associated macrophages. Px, Paxillin; Tn, Talin; TAM, tumor associated macrophages.

Integrins are expressed in an inactive confirmation on circulating immune cells [49,56,57]. Inflammatory factors released by tissues activate G protein coupled receptors (GPCRs), receptor tyrosine kinases (RTKs) or Toll-like receptor/interleukin1 receptor family members (TLR/IL1Rs), which initiate myeloid cell recruitment during inflammation [50]. We recently demonstrated that PI3Kγ promotes inflammation downstream of diverse receptors by stimulating inside-out activation of integrin, α4β1, granulocytic and monocytic cell adhesion to endothelium and invasion into tumors [51]. Pharmacological or genetic blockade of PI3Kγ suppressed adhesion and recruitment of monocytic and granulocytic cells into inflamed tissues. These findings suggested that targeting the trafficking of myeloid cells into tumors might provide significant benefit in the treatment of a wide variety of diseases. While all the steps in integrin activation have yet to be deciphered, PI3Kγ activates the small GTPase, Rap1, which promotes talin binding to integrin β1 − subunit cytoplasmic domains, thereby inducing a shift in the conformation of the extracellular domain of the integrin and increasing ligand binding affinity [58,59]. In addition, paxillin binding to the α4 cytoplasmic tail enhances integrin α4 activation, as disruption of the paxillin binding site in the integrin α4 cytoplasmic tail partially prevents talin binding and inhibits adhesion under flow *in vitro* and *in vivo* (Figure 1) [42,60,61]. 

## Roles of myeloid cells in tumor progression

### Angiogenesis

**Figure 2:**
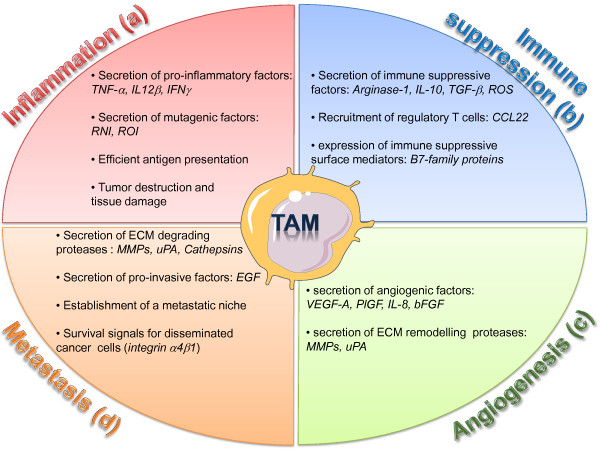
**Diverse roles of TAMs in the tumor microenvironment.** TAMs are critical regulator of tumor initiation and progression. (**a**) Inflammatory TAMs can initiate a chronic smoldering inflammation that creates a mutagenic and growth-promoting environment. (**b**) In established tumors, immune suppressive TAMs secrete factors which inhibit the activation of cytotoxic T cells and promotes the recruitment of regulatory T cells. (**c**) Pro-angiogenic TAMs stimulate the formation of new blood vessels by secreting angiogenic growth factors and ECM remodeling proteases. (**d**) TAMs support invasion and metastasis of malignant cells by destructing the ECM though proteases, by secreting invasion-inducing factors, and by supporting the establishment of a pro-metastatic niche. ROI, reactive oxygen intermediates; RNI, reactive nitrogen intermediates; ECM, extracellular matrix.

MDSC and TAMs play major roles in vascular remodeling during tumor progression. MDSC and TAMs release a number of potent pro-angiogenic cytokines, such as VEGF-A, VEGF-C, TNF-α, Placenta derived growth factor β (PlGF), chemokines (CXCL12, CXCL8), and bFGF [62,63]. TAMs also express a broad array of proteases known to play roles in the angiogenic process, including urokinase-type plasminogen activator (uPA), the matrix metalloproteinases MMP-2, MMP-7, MMP-9 and MMP-12 and elastase [64,65]. uPA and MMP support angiogenesis by remodeling and breaking down the extracellular matrix (ECM). Degradation of ECM leads to the mobilization of growth factors and facilitates the migration of vascular cells into new environments [66,67] (Figure 2c). 

### Immune suppression

MDSC and TAMs are both major regulators of the immune response [2].

MDSC suppress T cell proliferation in part by expression of Arginase-1 [68]. L-arginine plays a critical role in the inhibition of cytotoxic T cells by MDSC. Arginase converts L-arginine into L-ornithine and urea, thereby depleting L-arginine from the microenvironment and preventing iNOS from converting L-arginine to NO, an immunostimulant [69]. Depletion of arginine by Arginase I inhibits expression of the T-Cell Receptor (TCR) CD3zeta chain and T cell proliferation [70]. MDSC produced ROS also inhibits CD8^+^ T cell function by catalyzing the nitration of the TCR and thereby preventing T cell peptide-MHC interactions [71]. Moreover, several known tumor-derived factors, such as TGF-β, IL-3, IL-6, IL-l0, Platelet derived growth factor β, and granulocyte macrophage colony stimulating factor (GM-CSF) can induce the production of ROS by MDSC [8,72].

Beside inhibition of T cell activation, MDSC secrete immune suppressive cytokine with can inhibit immune surveillance. Secretion of the type 2 cytokine IL-l0 down-regulates the production of the type 1 cytokine IL-12 in macrophages. In addition, IL-l0 and VEGF-A inhibit the maturation of DC [68]. TGF-β has also been associated with MDSC immune suppressive functions. In fibrosarcoma and colon carcinoma tumor models, MDSC produced TGF-β in response to IL-13 stimulation, which resulted in decreased tumor immunosurveillance of cytotoxic T –cells [73,74].

### Myeloid cells in relapse or resistance to therapy

CD11b^+^Grl^+^ myeloid cells and TAMs play key roles in regulating the response of tumors to therapy, including anti-angiogenic and chemotherapeutic treatments. Accumulation of CD11b^+^Grl^+^ cells in tumors inhibits responsiveness to anti-angiogenic blockade by anti-VEGF-A antibodies [75]. Bv8, a protein expressed by myeloid cells in the bone marrow, stimulated the expansion and mobilization of CD11b^+^Grl^+^ cells in the bone marrow and mediated resistance to anti-VEGFA therapy [76,77].

### Macrophages and anti-cancer therapy

The significance of the vascular remodeling functions of TAMs in cancer therapy has recently emphasized by several studies. Tumor blood vessels are mostly disorganized and immature compared to non-pathological angiogenesis. Blood vessels are more torturous, with reduced pericyte coverage, and reduced erratic blood flow [78]. A recent studied showed that blood vessel normalization can be modulated by targeting the angiopoietin/Tie2 pathway. Interestingly, the angiopoietin receptor Tie2 is not only expressed on endothelial cells, but also a subpopulation of tumor infiltrating macrophages with vascular remodeling function. Targeting the Angiopoietin/Tie2 pathway by a fully humanized anti-ANG2 monoclonal antibody inhibited tumor angiongenesis, growth, and metastasis, and disabled the pro-angiogenic functions of tumor infiltrating macrophages, thus impeding the emergence of evasive resistance to anti-angiogenic therapy [79]. Genetic depletion of VEGF-A gene under the macrophage specific promoter LysM-Cre attenuates tumor angiogenesis and results in a morphologically more normal vasculature___. Tumors with normalized blood vessels showed increased sensitivity to chemotherapeutic treatment [80]. Similarly, histidine-rich glycoprotein HRG, a host-produced protein deposited in tumor stroma, can induce a reprogramming of the vascular remodeling functions of TAMs, resulting in vascular normalization and improved responses to chemotherapy [18]. In another report, blockade of CSF-1 signaling in a breast cancer tumor model, resulted in reduced numbers of intra-tumoral macrophage, normalized tumor vasculature, and increased responses to chemotherapy [47]. Notably, beside vascular normalization, both studies also showed enhanced anti-tumor immune responses, thus indicating the complexity of crosstalk’s by diverse cell types within the tumor microenvironment, and the power of targeting one subtype to thereby subvert biological functions of other stromal cells.

A mechanism independent of vascular normalization was proposed by Johanna Joyce and colleagues. The authors identified that TAMs secreted factors that protect tumors from chemotherapy. In the PyMT breast cancer models, tumors treated with the chemotoxic agent paclitaxel had more TAMs than control tumors. These TAMs expressed increased levels of proteases, specifically cysteine cathepsin B. Expression of cathepsin B was suggested to be necessary to protect cancer cells *in vitro* and *in vivo* from several chemotoxic agents, including paclitaxel, etoposide, and doxorubicin [81].

## Conclusions

Myeloid cells promote tumor progression and alter the response of tumors to anti-cancer therapies. Identification and targeting of myeloid cells represents an emerging and attractive therapeutic approach to fight cancer. Therapeutic strategies targeting TAMs include inhibition of their recruitment to the tumor microenvironment, blockade of their pro-tumoral effector functions, and reprogramming of macrophage/MDSC polarization and thus restoring their anti-tumorigenic functions. Targeting myeloid cell recruitment can reduce tumor progression and improve the efficacy of chemotherapeutic treatments [41,51]. Similarly, partial reprogramming of macrophage polarization towards an M1-like phenotype enhances chemotherapy and reduces tumor growth [18,82]. Importantly, some of the anti-tumorigenic functions of macrophages critically depend on the presence of cytotoxic CD8^+^ T-cells, which are part of the adaptive immune system [47,83].

## Competing interests

Both authors declare that they have no competing interests.

## Authors’ contribution

MCS and JVS reviewed the literature on myeloid cells in tumor inflammation, prepared the manuscript, and drew the illustrations. All authors read and approved the final manuscript.
